# Disentangling vehicular emission impact on urban air pollution using ethanol as a tracer

**DOI:** 10.1038/s41598-018-29138-7

**Published:** 2018-07-16

**Authors:** Joel Brito, Samara Carbone, Djacinto A. Monteiro dos Santos, Pamela Dominutti, Nilmara de Oliveira Alves, Luciana V. Rizzo, Paulo Artaxo

**Affiliations:** 1 Institute of Physics, University of São Paulo, São Paulo, Brazil; 2 Laboratory for Meteorological Physics (LaMP), Université Clermont Auvergne, F-63000 Clermont-Ferrand, France; 3 Federal University of Uberlândia, Uberlândia, MG Brazil; 4 Department of Pathology, School of Medicine, University of São Paulo, São Paulo, Brazil; 5 Departamento de Ciencias Ambientais, Universidade Federal de São Paulo, São Paulo, Brazil

## Abstract

The Sao Paulo Metropolitan Area is a unique case worldwide due to the extensive use of biofuel, particularly ethanol, by its large fleet of nearly 8 million cars. Based on source apportionment analysis of Organic Aerosols in downtown Sao Paulo, and using ethanol as tracer of passenger vehicles, we have identified primary emissions from light-duty-vehicles (LDV) and heavy-duty-vehicles (HDV), as well as secondary process component. Each of those factors mirror a relevant primary source or secondary process in this densely occupied area. Using those factors as predictors in a multiple linear regression analysis of a wide range of pollutants, we have quantified the role of primary LDV or HDV emissions, as well as atmospheric secondary processes, on air quality degradation. Results show a significant contribution of HDV emissions, despite contributing only about 5% of vehicles number in the region. The latter is responsible, for example, of 40% and 47% of benzene and black carbon atmospheric concentration, respectively. This work describes an innovative use of biofuel as a tracer of passenger vehicle emissions, allowing to better understand the role of vehicular sources on air quality degradation in one of most populated megacities worldwide.

## Introduction

Large urban conglomerates are well-known air pollution hotspots, with impacts ranging from local air quality degradation^1^ up to global climate^2,3^, where emission from the transportation sector plays a pivotal role^4^. Within the Sao Paulo Metropolitan Area (SPMA), passenger vehicles outnumber buses by over 100 to 1, and trucks by over 30 to 1. Nonetheless, on a vehicle basis, buses and trucks emission of pollutants such as nitric oxide (NO) and particulate matter tends to exceed those of passenger vehicles by roughly the same order of magnitude^5–7^. Furthermore, distinct vehicle circulation patterns lead to heterogeneous spatial and temporal air pollutant emissions. Combined with a complex atmospheric chemistry and dynamics, the identification and quantification of the role of vehicle types on air quality remains largely an open issue^8–10^.

In 1975, the Brazilian government created a national program to stimulate the use of ethanol as vehicle fuel, which mandated its mixture in gasoline. Since then, ethanol penetration in Brazil has largely varied throughout the years, depending not only on available vehicle technology, but also on the price of oil, the price of sugarcane derivatives (e.g. sugar), government incentives and so forth. In early 1990s there was a peak in hydrous ethanol (E100) fuelled passenger cars within the SPMA, which accounted for half of the fleet, whereas the other half was gasohol fuelled (with 25% ethanol mix in gasoline, E25)^11^. In 2003, with the introduction of flex-fuelled passenger vehicles, consumers were able to choose at the petrol station any mixture of ethanol between E25 and E100. Around the world, there is a tendency of increase of biofuel consumption by the transportation sector^12^, nonetheless its global penetration remain fairly small. For a basis of comparison, in 2013 (the time of our measurements) average fuel consumption by passenger vehicles within SPMA amounted to 55% ethanol and 45% gasoline (on fleet-wide average, the equivalent of an E55 fuel). Conversely, in the UK the fraction of ethanol is about 5% (E5) and expected to rise in the near future to E10, a comparable fraction to other countries in the world.

Given that ethanol is used as fuel uniquely by Light Duty Vehicles (LDV, cars and motorcycles), in contrast to diesel fuelled Heavy Duty Vehicles (e.g. buses and trucks), we propose its use to disentangle traffic emissions on a number of atmospheric pollutants. To our knowledge, this study represents the first use of ethanol as a real-time tracer of passenger car emissions on a source apportionment analysis. As biofuel use continue to rise worldwide^12^, atmospheric ethanol concentration should prove a valuable tracer in understanding the primary emission of different fractions of transportation sector in urban environments. In times when understanding air pollution health effects are becoming deeper and wider^13^, policy makers should be presented clear paths to improve urban air quality.

## Results and Discussion

The range of atmospheric measurements conducted in downtown Sao Paulo is described in Table 1. The basis of our study is the organic aerosol source apportionment analysis based on Positive Matrix Factorization (PMF) on nearly real-time Aerosol Mass Spectrometry measurements (Table 2)^14^. This type of analysis allows the identification of some primary sources (e.g. traffic^15^, cooking^16^, biomass burning^17^) and secondary processes (such as particulate matter formation via oxidation of isoprene^18^). The PMF factors identification is possible via spectral signatures analysis and correlation with known tracers (e.g. Carbon Monoxide, CO, for traffic or Ozone for secondary processes through photochemistry). The use of ethanol as tracer for factor identification is discussed in the following.

**Table 1:** Description of the different data sets used in this study and summary statistics. Measurement data has been averaged into 1-h bins, resulting in 741 valid data points from 08 February to 08 April 2013.

Variable and unit of measurement	Method	Data source	Median (and IQ)
PM_10_ mass concentration (µg m^−3^)	Beta continuous	CETESB	28 (19–40)
PM_1_ mass concentration (µg m^−3^)	ACSM + MAAP	Own	10.80 (7.49–16.31)
BC mass concentration (µg m^−3^)	MAAP	Own	2.71 (1.55–4.27)
Aerosol particle larger than 7 nm (N) number concentration (10,000 cm^−3^)	DMPS	Own	1.36 (1.03–1.71)
CO mixing ratio (ppm)	IR Photometry	CETESB	0.6 (0.4–0.9)
NO mixing ratio (ppb)	Chemiluminescence	CETESB	23 (7–47)
Ozone mixing ratio (ppb)	UV Photometry	CETESB	21.9 (13.2–30.9)
Ethanol mixing ratio (ppb)	PTRMS	Own	24.20 (20.35–29.52)
Acetaldehyde mixing ratio (ppb)	PTRMS	Own	3.25 (2.28–4.33)
Benzene mixing ratio (ppb)	PTRMS	Own	0.58 (0.41–0.87)
Toluene mixing ratio (ppb)	PTRMS	Own	1.59 (0.99–2.59)

**Table 2:** Concentration of PMF factor loadings and ambient concentration of chemical species in the submicrometric size range. Measurement data has been averaged into 1-h bins, from similar statistics as Table 1.

Variable and unit of measurement	Median (and IQ)
Factor LDV-OA (µg m^−3^)	1.22 (0.73–1.80)
Factor HDV-OA (µg m^−3^)	0.85 (0.44–1.60)
Factor OOA-I (µg m^−3^)	0.68 (0.36–1.15)
Factor OOA-II (µg m^−3^)	1.57 (0.78–2.78)
Organic Aerosol (µg m^−3^)	4.91 (3.26–7.43)
Sulphate (µg m^−3^)	1.64 (1.14–2.38)
Nitrate (µg m^−3^)	0.44 (0.24–0.96)
Ammonium (µg m^−3^)	0.66 (0.33–1.13)

### Ethanol is observed in high concentrations in downtown Sao Paulo and serves as tracer of LDV primary emission

Ethanol has been observed in downtown Sao Paulo in concentrations ranging from 20 to 30 ppbv (Table 1). This concentration range, although high, is lower than has been reported in downtown Sao Paulo some decades ago (average of 170 or 470 ppbv depending on the sampling site)^19,20^. The decrease in ethanol concentration (despite the steep increase in vehicles number) is associated to both engine technology improvement, as well as increased use of gasoline/ethanol mixture in lieu of pure hydrous ethanol (E100)^11^. To put those concentrations in context, in places where ethanol is not a vehicular fuel additive, typical remote/rural background concentration is in the range of 0.02 to 0.4 ppbv^21–24^. Urban measurements in Pittsburgh, in early 2000s, reported ethanol values ranging from 0.6 to 3.5 ppbv^25^, largely associated to industrial sources with a minor contribution of biogenic emissions^24^. More recently, in London, where gasoline currently contains 5% of ethanol (E5), ethanol has been reported as the most abundant Volatile Organic Compound (VOC), with an average mixing ratio of 5 ppbv^26^. Conversely, average ethanol concentration in 2010 in the Los Angeles Basin was reported at 9 ppbv, mostly associated to use as fuel additive (typically E10), a stark increase in concentration relative to few years prior^27^. Such increase is also expected in other urban areas where ethanol usage is on the rise.

**Figure 1:**
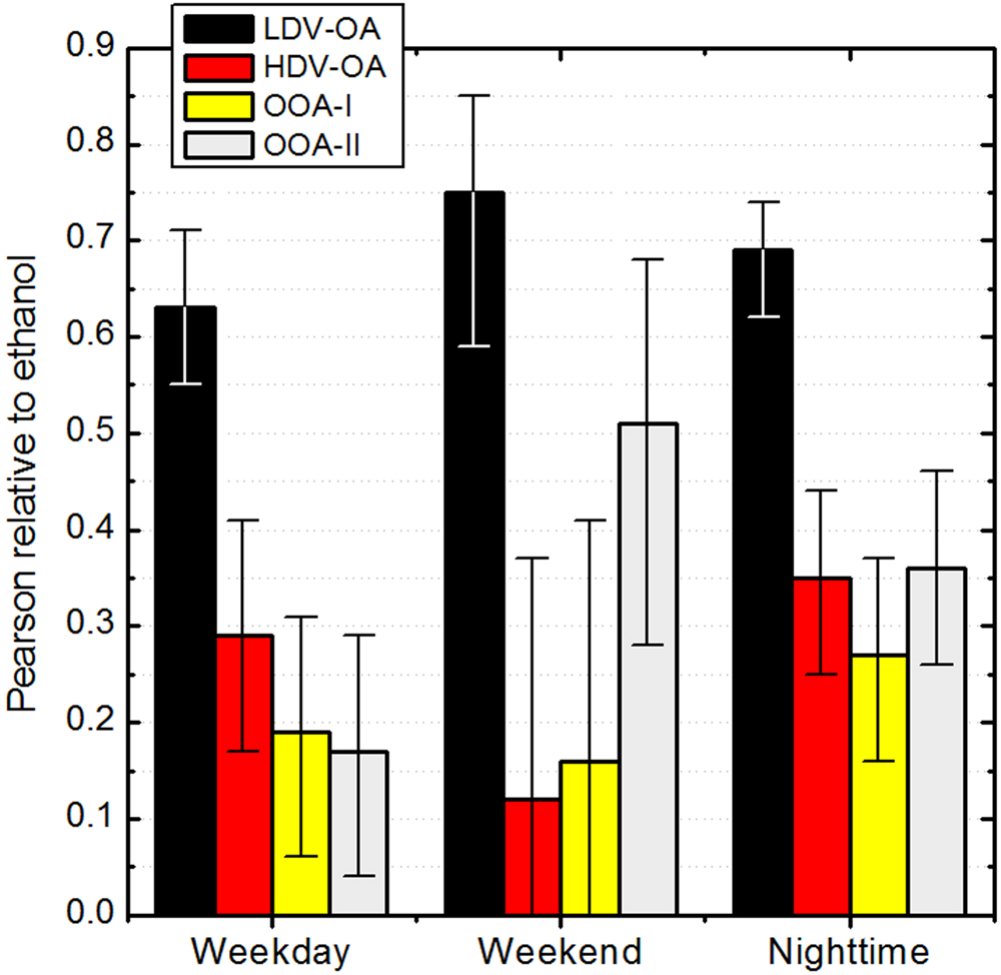
Linear fit Pearson correlation between ethanol and factors of organic aerosols resolved using PMF. Weekday (Monday thru Friday) and Weekend (Sunday only) refers for daytime data (06–18:00 LT) and Nighttime refers to 18:00–06:00 LT without day of the week separation. Range bars indicate confidence intervals. All fits are statistically meaningful (p < 0.005) except weekend data of HDV-OA and OOA-I.

The PMF analysis conducted here has identified two Organic Aerosol (OA) factors containing typical traffic spectral signature, and two other factors linked to secondary processes, termed Oxygenated Organic Aerosol (OOA) I and II, the latter being more oxidized (typically more processed) than the former. Figure 1 shows linear fit results between ethanol ambient concentration and factor loadings, where the factor depicting high correlation with ethanol has been termed Light-Duty-Vehicle Organic Aerosol (LDV-OA), and the other is Heavy-Duty-Vehicle Organic Aerosol (HDV-OA). An advantage of the use of ethanol as tracer for LDV emissions arises from fairly short lifetime (2.8 days)^24^, and in comparable range as the traffic-related organic aerosol factor (1–3 days)^28,29^. Nonetheless, it is important to note that ethanol tailpipe emission varies strongly throughout the driving cycle, being significantly high during engine cold start, levelling off after a few minutes^30^. In addition, given its high volatility, a fraction of ethanol originates from evaporation (and thus will not be co-emitted with combustion emitted LDV-OA). Thirdly, ethanol measurements described here can suffer from a (minor) interference from formic acid^31^, further discussed in the methodology section. Taken those three aspects together, one does not expect a perfect correlation between LDV-OA loading and ethanol ambient concentration. Despite those caveats, the high ambient concentration and its significant correlation with a traffic-related OA factor supports its use as tracer of LDV emissions. It is important to note that observations were obtained from a single sampling site, and thus its results can be considered representative only of the surrounding (i.e. downtown) area. Furthermore, PMF analysis are known to be affected by uncertainties such as random errors and rotational ambiguities^14^, which were minimized by following state-of-the-art analysis procedures (Material and Methods).

**Figure 2:**
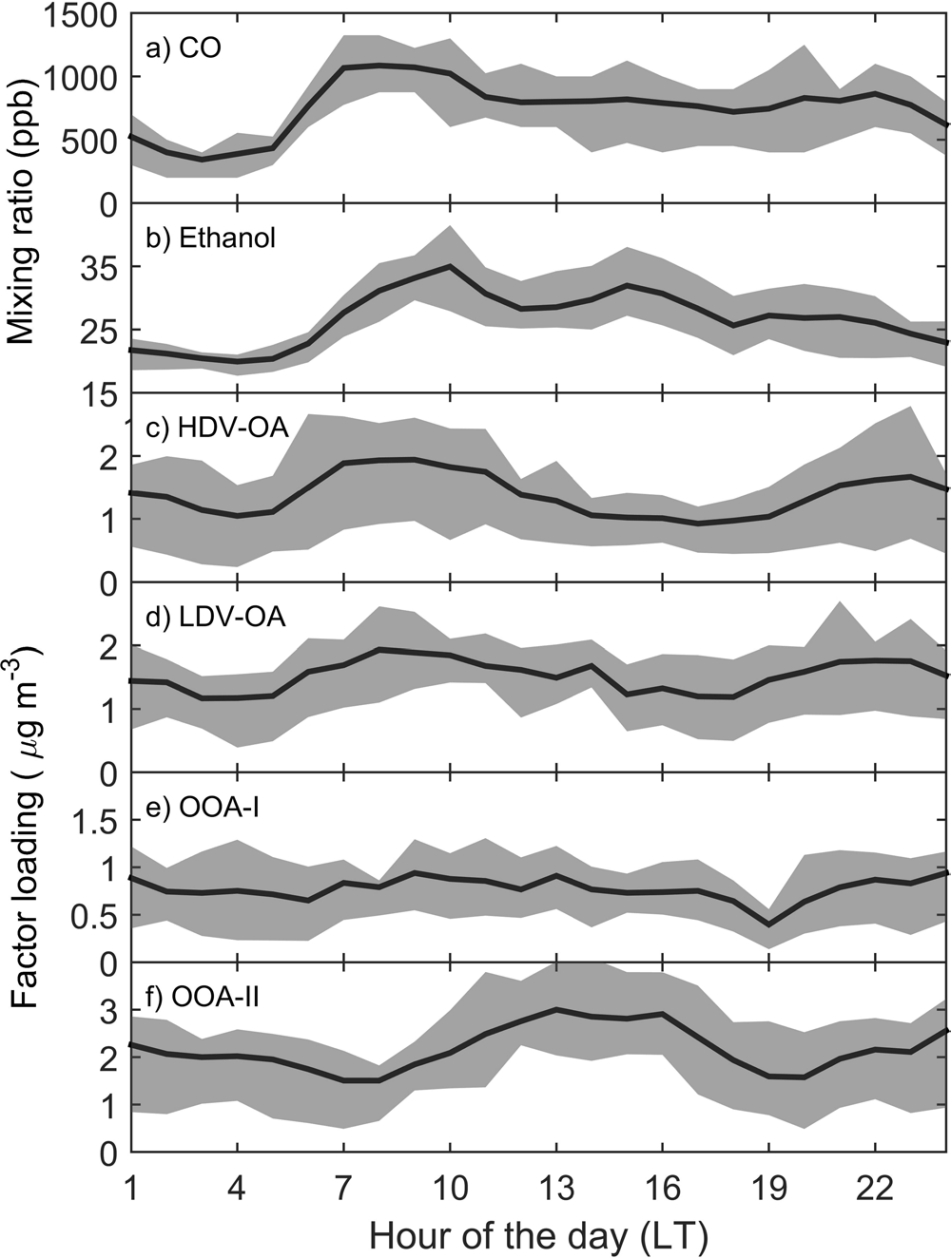
Weekday diurnal profile of CO (**a**), ethanol (**b**), HDV factor (**c**), LDV factor (**d**), OOA-I factor (**e**), OOA-II factor (**f**). Black line represents median and grey area the interquartile range.

The diurnal variability during weekdays is shown in Fig. 2, with CO, ethanol, HDV-OA and LDV-OA modulated by rush-hour traffic emissions and atmospheric dynamics, such as boundary layer height and wind patterns. In contrast to the primary pollutant patterns, OOA-I and OOA-II depict a different diurnal variability, whether slightly constant concentration throughout the day, or significantly increasing in the afternoon, in line with similar source apportionment studies conducted in urban environments^14,32^. The factor with highest contribution to OA concentration is OOA-II (Table 2), a typical result from urban environments^28,33^, followed by LDV-OA, HDV-OA, and OOA-I. It is interesting to note that the estimates of primary (LDV-OA and HDV-OA) and secondary (OOA-I and OOA-II) organic aerosols obtained here are remarkably close to a recent study at the same sampling site via offline filter analysis^34^. By applying a OC:OA ratio of 1.2 and 1.6 for primary and secondary OA components^35^, our primary OC (LDV + HDV) average would be 1.7 µg m^−3^ versus filter estimation of 1.6 µg m^−3^ whereas secondary OC (OOA-I + OOA-II) would be 1.5 µg m^−3^ and filter estimates were 1.4 µg m^−3^.

The impact assessment of primary LDV and HDV emissions and secondary processes on ambient concentration of a range of pollutants in downtown Sao Paulo is achieved by using PMF factor loadings as predictors in a multivariate linear regression of a range of pollutants atmospheric concentration. The main advantage of this approach is that PMF factor loading (as the pollutant concentration) is the convolution between emission strength and subsequent atmospheric dispersion/processing. Therefore, by using PMF factors as predictors, the methodology intrinsically takes into account complex atmospheric dynamics between source emission and measurement, which can otherwise require an extensive amount of input data to properly parametrize (e.g. Salvo *et al*.^36^). Conversely, a limitation of the methodology proposed here is to force species of interest to be described by the four PMF factors (lacking, for example, an industry factor as predictor). It is well-known, nonetheless, that traffic emissions are the main pollution source within SPMA^11^. For example, in the 1980s and 1990s, many industries relocated from SPMA to other regions towards less stringing pollution emission regulations, significantly reducing industrial emissions contribution to air pollutants in Sao Paulo. In a background site of SPMA, a recent particulate matter source apportionment study has identified only a minor contribution of industrial (9.5%) and biomass burning (7.6%) emissions^37^. Our sampling site in downtown SPMA, surrounded by 12–35 km of highly dense urban occupation^38^, is likely to be even less impacted by those sources.

**Figure 3:**
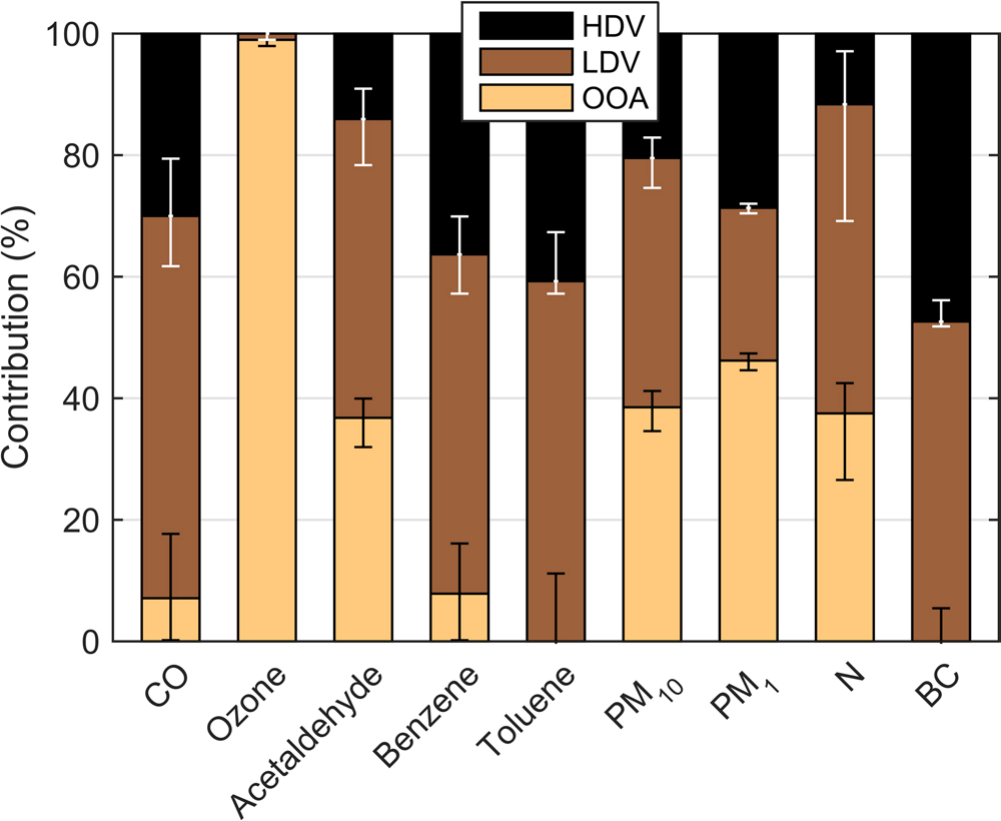
Relative contribution of primary (LDV, HDV) and secondary sources (OOA, the sum of OOA-I and OOA-II). Range bars represent 5^th^ and 95^th^ confidence interval.

Our main results are shown in Fig. 3, namely the estimated role of vehicle type on several pollutants ambient concentration in downtown Sao Paulo. The results presented here were filtered for pollutants yielding adjusted R^2^ above 0.75. Pollutants regression results were analysed according to their typical atmospheric residence time and compared against emission inventories and/or literature estimates, when available.

### CO, acetaldehyde, toluene and benzene are mostly apportioned to LDV emissions

According to our analysis, about 65% of CO measured at the downtown site is attributed to LDV emissions (Fig. 3). This is in general agreement with municipal official inventories apportioning passenger vehicles about 59% of total CO emission^7^, and an extra contribution of 26% of gasohol fuelled motorcycles. It is important to note that chemical lifetime of CO is significantly longer than LDV-OA (>3 months^39^ compared to a few days^28,29^, respectively), which can explain why a fraction (6%) of CO is also associated to aged air masses (the OOA factors).

Acetaldehyde is an important oxygenated VOC with well-established impacts on human health^40^ and ozone formation^41^. Furthermore, acetaldehyde tailpipe emission is known to scale with ethanol content in gasoline^42^, and is one of secondary products of ethanol atmospheric oxidation^43^, being typically considered one of the most important drawbacks of biofuel use in terms of urban air quality^43,44^. Our analysis apportioned 49% and 14% of acetaldehyde concentration to LDV and HDV tailpipe emission, respectively. Secondarily formed acetaldehyde would be 37%, which is likely to originate from ethanol atmospheric oxidation at this site^38^. It is important to note that despite the high ethanol use from LDV, tunnel measurements in the outskirts of SPMA have recently reported acetaldehyde emissions factors (per km) of heavy transport trucks to be three times higher than passenger vehicles (7.4 and 20 mg km^−1^, respectively)^45^. Nonetheless, by assuming that secondary acetaldehyde also originates from LDV emissions (through ethanol oxidation), the latter would be responsible for about 85% of acetaldehyde ambient concentration in this downtown site. Ozone, an important secondary pollutant under urban environments^39^, is entirely attributed to OOA (Fig. 3), a well-established relationship^14^, thus mostly serving as corroboration of the methodology described here.

Benzene and toluene are also VOCs with important effects on human health^46^ and relevant aerosol precursors^47^. Results show that about 60% of both species are attributed to LDV emissions, whereas the remaining is mostly HDV. Whereas chemical lifetime of toluene (1.9 day)^41^ is comparable to LDV-OA, benzene is somewhat higher (9.4 day)^41^, which can explain why there is a fraction of the latter that is also attributed to aged air masses (the OOA factors), similarly as CO.

### PM_10_ is dominated by LDV and OOA

The composition of particulate matter less than 10 µm (PM_10_) is known to be highly heterogeneous, with a typical atmospheric residence time from a few days to some weeks^39^. Whereas the apportionment analysis described here rely solely on the temporal co-variability with organic aerosol factors, PM_10_ sources can also be apportioned through its chemical composition. A recent study in a background site in SPMA, based on offline chemical composition PM_10_ analysis, has identified that road dust and vehicular sources contributed 26% and 39%, respectively^37^. Road dust in urban environments (particularly SPMA, far from desert areas) are typically associated to resuspension caused by traffic circulation^37^. Furthermore, the MLR analysis presented here (using HDV-OA, LDV-OA and OOA as predictors) has explained more than 92% of PM10 variability (Table S1), indicating little contribution of other sources than traffic (HDV/LDV) or secondary/long-range transport (OOA). Conversely, the results from Pereira *et al*. (2017)^37^ show a combined contribution of dust and traffic to PM10 of 64%, in good agreement with our analysis (Fig. 3, 41% LDV + 20% HDV = 61%). Nonetheless, whereas PM_10_ attributed to OOA here certainly includes secondary species, it is also likely to account for aged air masses (e.g. intrusion of regional pollution into SPMA). The official particulate matter emissions inventory of SPMA attributes only 7% to LDV emissions, whereas city transit buses and urban cargo vehicles would account for roughly 30%^7^. However, the inventory considers only direct tailpipe emission, disregarding break dust, tyre wear and resuspension, which is known to comprise a significant fraction of PM_10_ from traffic^6,48^.

### PM_1_ and BC have comparable contribution of LDV and HDV

Approximately half of of particulate matter less than 1 µm (PM_1_) in downtown Sao Paulo is equally attributed to primary emission of LDV and HDV (Fig. 3). The non-primary fraction of PM_1_, being the largest component, is mostly attributed to secondary formation as can be observed via its diurnal variability (Fig. 2) and contribution of secondary species, such as sulphate (Tables 1 and 2). The significant contribution of secondary process on fine particulate matter is in line with other megacities studies worldwide^33^. It is important to note that a significant component of the inorganic component of PM_1_, particularly sulphate, is likely to be mainly attributed to HDV, given the relatively high sulphur content in diesel at the time of the campaign (up to 1800 ppm, has been decreased nowadays to 500 ppm, with existing option down to 10 ppm). Nonetheless, adjusted R^2^ for sulphate has been lower than other components (R^2^ = 0.67, Supplementary Table S1), suggesting contribution from other sources (e.g. industrial) or too distinct atmospheric processing relative to OOA. As for the secondary organic aerosol, the role of diesel versus gasoline has been the subject of strong debate, with conflicting results from molecular and ambient analysis^49^, and largely remains an open issue.

Black carbon is equally apportioned to LDV and HDV emissions, without a contribution of OOA factor, indicating to be mostly locally emitted (in contrast to being advected into the area). Tunnel measurements have obtained an HDV BC emission factor almost 30 times those of LDV^50^, however recent roadside calculation of BC emission factors of HDV and LDV within Sao Paulo has estimated the former to be only four times the latter^51^, in strong contrast to previous tunnel measurements. Considering the comparable contribution of HDV and LDV emissions to BC presented here, and the number of vehicles in the vicinity of our sampling site (Supplemental material), we estimate a per vehicle BC emission of HDV to be roughly 23 times those of LDV. Therefore, our results are in line with tunnel measurements (HDV BC emission factor being a factor 30 relative to LDV)^50^ rather than ambient estimates (a factor of four only)^51^.

### LDV and OOA have largest contribution to aerosol number concentration

Aerosols in urban environments are mostly found in the ultrafine range (<50 nm)^52^, with significant potential for lung deposition and translocation to other parts of the body^53^. Those ultrafine particles (UFP) are typically result of both primary emission (e.g. traffic) and secondary gas-to-particle conversion resulting in new particles formation events^52^. Tunnel aerosol characterization in the outskirts of SPMA, for example, has identified an average diameter associated with LDV and HDV of 48 nm and 39 nm, respectively^6^. Conversely, a three-month field campaign in a background site within SPMA has identified secondary new particle formation events in 11% of the days^54^. Our analysis apportions about half of aerosol number particle concentration above 7 nm (N) to LDV primary emissions, 37% to secondary processes and 12% to HDV emissions (Fig. 3). The significant contribution of LDV to aerosol number concentration obtained here corroborates a recent study, which has observed a significant effect on UFP in a background sampling site in SPMA associated to the ethanol content used by passenger vehicles^36^.

### Implications for air quality policies

It is well-established that vehicular emissions have a pivotal role in air quality degradation in the SPMA^11,36–38^. By the time of our field campaign, official records show about 8.8 million gasohol-fuelled vehicles (cars and motorcycles), whereas diesel fuelled vehicles amounted to some 0.5 million vehicles (220 000 heavy trucks, 110 000 buses/minibuses and 95 000 urban cargo vehicles, among others)^55^. In addition to the extensive local fleet, SPMA is a large road transport hub, with a significant number of heavy transport trucks crossing its outer beltway (or within the city during nighttime).

The Brazilian vehicular emission standards (termed PROCONVE, Program for the Control of Air Pollution Emissions by Motor Vehicles) dictates for the past 30 years emission factor standards for LDV (now in phase 6) and HDV (phase 7). Prior the implementation of the program (and even during its early phases), yearly average CO concentration often reached about 20 ppmv, and has been steadily decreasing since 2000s to reach about 1 ppmv in recent years^11^. For the same period, pollutant reduction for other species was less efficient, with PM_10_ decreasing from 80 µg m^−3^ to about 30 µg m^−3^ and ozone daily maxima keeping steady at about 120 ppbv for the past 30 years^11^. Current regulatory emission factors tend to follow relatively close European emission standards, e.g. Euro 6 CO emission for passenger vehicles is 1.0 g km^−1^ whereas PROCONVE phase 6 is 1.3 g km^−1^. Whether further reducing emission factors is certainly desired, it becomes increasingly challenging in terms technological/cost requirements, especially the significant shift to secondary (and often non-linear) processes often dominating air quality^33^.

The results here show a significant contribution of HDV primary emissions to most pollutants, ranging from about 14% (acetaldehyde) up to 46% (BC) despite being significantly outnumbered by passenger vehicles. According to our model, a 10% reduction of the number of LDV (HDV) emission would translate into a decrease of about 0.16 ppbv (0.04 ppbv) of acetaldehyde, 0.03 ppbv (0.02 ppbv) of benzene, 1.18 µg m^−3^ (0.59 µg m^−3^) of PM_10_, 0.31 µg m^−3^ (0.36 µg m^−3^) of PM_1_, 647 (147) aerosol number concentration and 0.17 µg m^−3^ (0.16 µg m^−3^) of BC. It is important to note that some of these estimates are lower bounds by not considering the effect of the secondary component (particularly relevant for acetaldehyde and particulate matter). Furthermore, our field campaign took place in a relatively unpolluted period of time, with efficient pollutant dispersion and removal by precipitation^38^, therefore such gains in emission reduction shall be significantly higher during dry, stagnant meteorological conditions typical of wintertime.

In addition to recommendation to scrap old vehicles (>10 years of age)^11^, and expanding the metro grid, air quality improvement recommendations can be provided based from the results presented here. It is likely that significant improvement (and best cost-effective) would be to further restrict passenger vehicle circulation in the downtown area (reducing overall LDV emissions) coupled with reduction of transit buses emission factors (filters, fuel quality, etc.). Through the application of similar instrumentation of described here, and using ethanol as tracer of LDV emission, the effects of such air quality improvement policies should be quantifiable, and thus serve as guide to further mitigate deleterious effects of traffic emissions on its 20 million inhabitants.

## Materials and Methods

### Field campaign

The measurements were conducted from 08 February to 08 April 2013 at the Public Health Faculty (23° 33′ 13.87″ S, 46° 40′ 23.46″ W), at the crossing of Dr. Arnaldo and Teodoro Sampaio avenues, in Sao Paulo downtown. The measurement of VOCs, non-refractory chemically speciated submicrometric aerosols, BC and aerosol number concentration were conducted at the third floor of the Public Health building, roughly 15 m above street level. Measurement points were averaged to 1 h time resolution and only included in the dataset if all instruments were operational (i.e., no missing data points), for comparable analysis.

### Aerosol and gas sampling

Air was transferred for gas-phase sampling through an unheated 5 m long 1/8 in. O.D. PTFE tube to the sampling system. Aerosol sampling was conducted using a 1.5 m long 1/4 in. O.D. copper tubing with a sample flow of 16.7 lpm, through a 50% diameter cut-off of 2.5 µm and diffusion drier (<50% RH). The hourly concentration of ozone, NO, CO and PM_10_ was provided by the São Paulo State Environmental Agency (CETESB, Cerqueira César station), some 50 m from the sampling site.

### Proton-Transfer-Reaction Mass Spectrometry (PTR-MS)

A quadrupole PTR-MS (Ionicon Analytic, Austria) was used to provide VOC mixing ratios with a time resolution of 1 min. The system was operated under standard conditions (2.2 mbar drift tube pressure, 600 V drift voltage, E/N 140 Td). Every 60 min air was diverted through a catalytic converter (Supelco, Inc. with platinum pellets heated to 400 °C) to assess the background signal for each species. A system calibration was performed during the campaign using permeation tubes stabilized at 40 °C for benzene and toluene^56^. Furthermore, both species and acetaldehyde were postcampaign calibrated using a set of gravimetrically prepared multicomponent mixtures (Apel Riemer Inc., USA) for a large range of humidity values. Concentration estimates of compounds calibrated by both methods agreed well within instrumental accuracy. Ethanol concentration was estimated from m/z 47, with calibration performed prior to the field campaign by Ionicon under similar operation conditions as applied here. It is important to note that ethanol measurements suffer from sensitivity issues, mainly attributed to fragmentation from H_3_O^+^ and interference with formic acid^31^, which makes its quantification under typical background conditions (e.g. biogenic only) challenging^27^. Similarly as other studies (e.g. Seco *et al*.^57^), our sensitivity for ethanol is also relatively low (3.22 ncps ppbv^−1^), in stark contrast to other species (e.g. methanol is 21.96 ncps ppbv^−1^). Nonetheless, given the high abundance of this species in downtown Sao Paulo^20^, the relatively low sensitivity does not affect data quality. Furthermore, formic acid concentrations measured at a Sao Paulo downtown site under similar meteorological conditions several years prior our field campaign yielded a formic acid concentration of 2.6 ppb^58^ (about only 10% of estimated ethanol concentration here). Considering a comparable concentration at the time of the campaign, it does not impact ethanol concentration estimates significantly, and much less undermines the identification of the LDV-OA factor.

### Aerosol measurements

An Aerosol Chemical Speciation Monitor (ACSM, Aerodyne Research Inc., USA) was used to provide real-time (30 min resolution) chemically resolved mass concentrations of particulate ammonium, nitrate, sulphate, chloride, and organic species in the submicron size range^59^. Mass calibration was conducted for 300 nm ammonium nitrate aerosols. The collection efficiency of the instrument has been calculated according to Middlebrook *et al*.^60^, typically yielding 0.5, being corroborated by collocated submicrometric aerosol volume measurements. The concentration of BC was measured using a Multiangle Absorption Photometer (MAAP, Thermo Scientific model 5012). Aerosol number concentration has been integrated from a Differential mobility particle sizer (DMPS, 7–800 nm). Measurements were validated against an independently operated condensation particle counter (CPC, model 3022, TSI Inc., St. Paul, MN, USA), for aerosol concentration below 10^4^ cm^−3^, a range which CPC counts aerosol individually and thus depicts smaller uncertainties.

### Organic aerosol source apportionment

**Figure 4:**
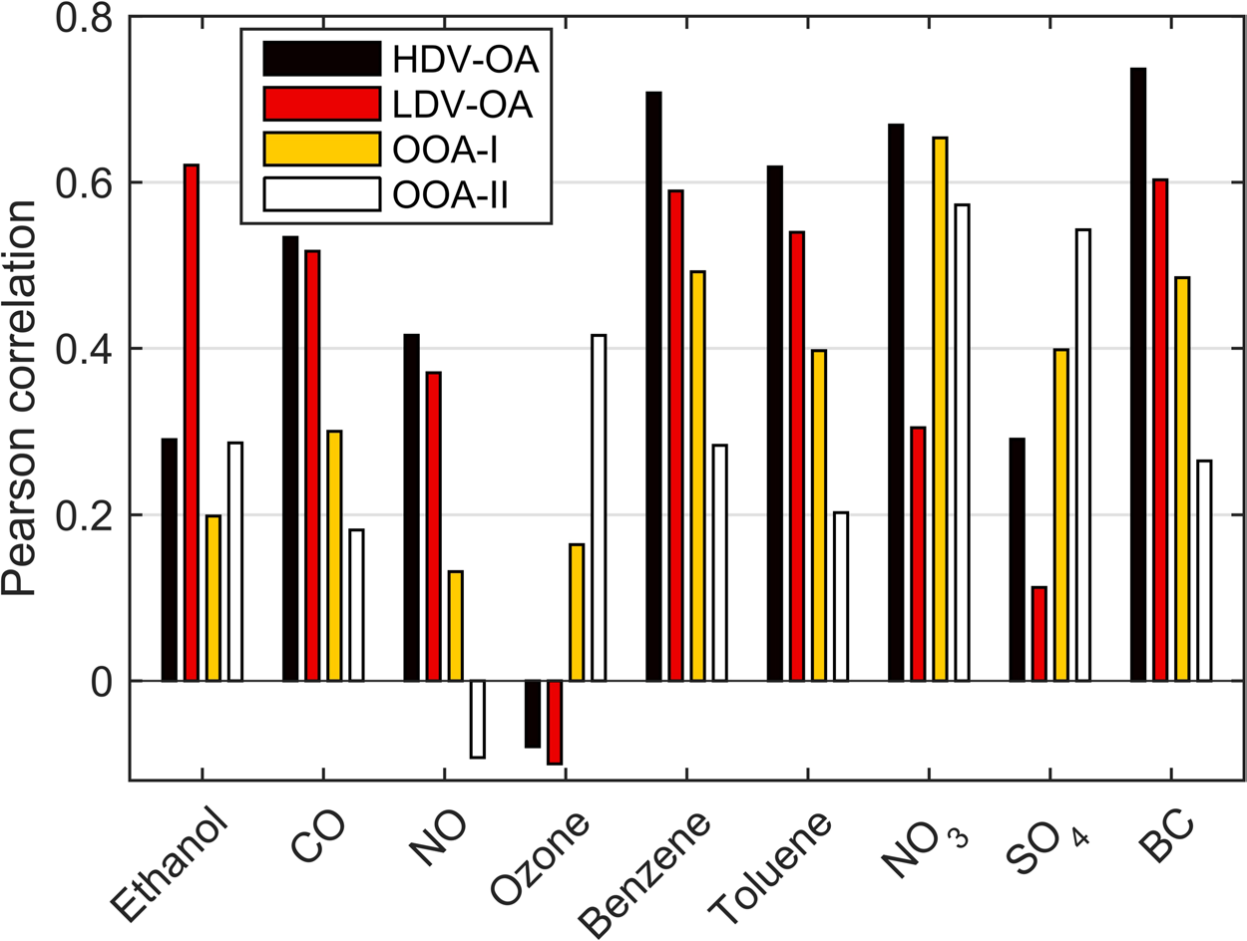
Pearson correlation between external tracers and PMF factors.

**Figure 5:**
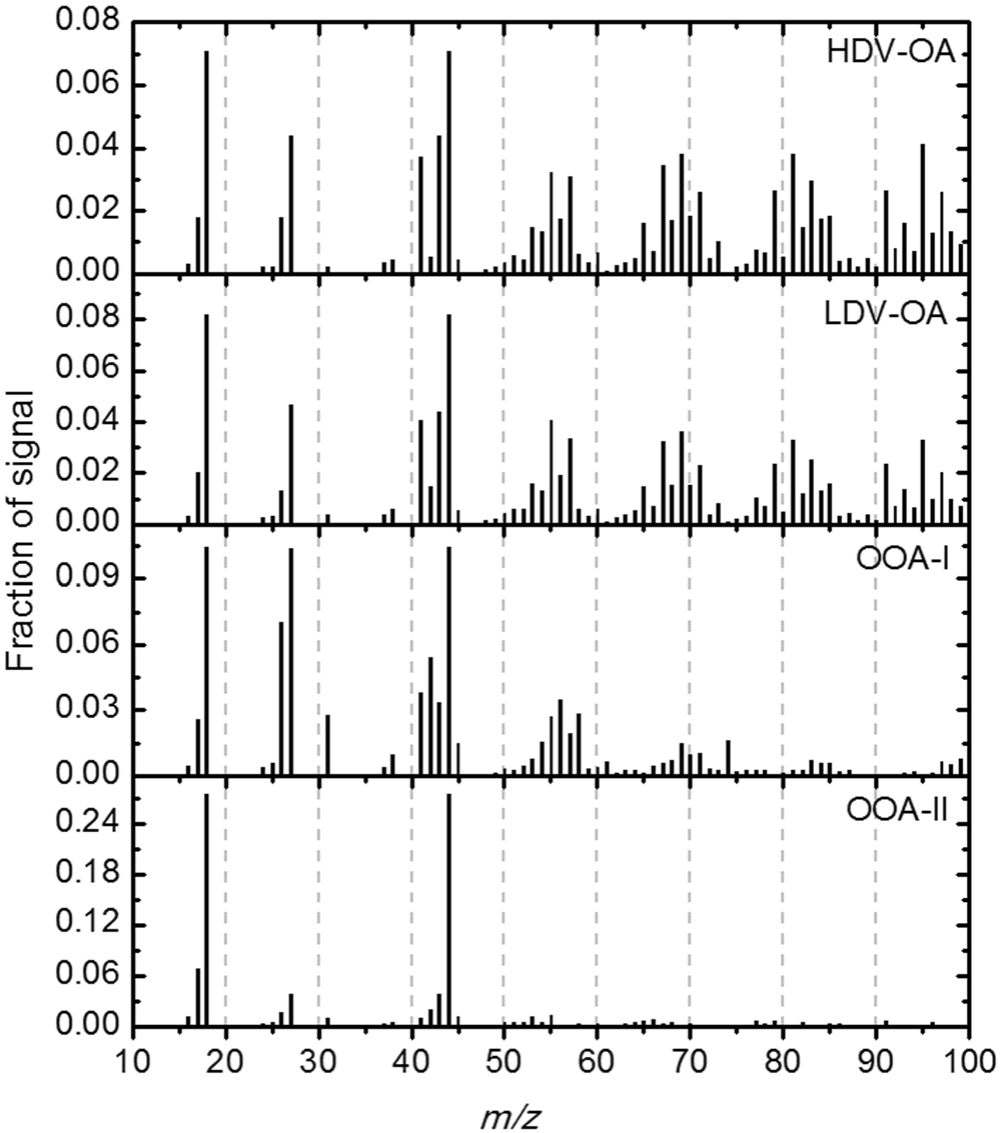
Mass spectra of identified factors of organic aerosol PMF analysis.

Positive matrix factorization of OA has been conducted following the procedure as described by Ulbrich *et al*.^61^, where factor classification is conducted based on correlation analysis with known external tracers (Fig. 4), spectral analysis (Fig. 5) and their diurnal variability (Fig. 2 and S2). Detailing of the PMF procedure (residual depending on number of factors and fpeaks) is provided in the supplemental material.

### Multiple Linear Regression

The Multiple Linear Regression analysis has been performed using OriginPro 8, with the PMF factor loadings as predictors and species of interest as dependent variable. For this analysis, intercept was set as 0, as it holds no physical significance in the source attribution. The contribution of each factor to pollutant concentration has been calculated for regressions with adjusted R^2^ higher than 0.75, and using regression parameters and campaign averages (Supplementary Table S1). Confidence intervals were calculated according to regression results upper and lower limits.

### Vehicle counts

The number of vehicles in the vicinity of the sampling site has been obtained from the 2013 road traffic performance from the Sao Paulo Traffic Engineering Company (CET)^62^. The reports describes a week worth of vehicular counts (13–21 October 2013) during commuting hours (morning 07:00–10:00 and evening 17:00–20:00) at the road transect at Dr. Arnaldo Avenue (Route 13G in the report), yielding average vehicle counts of 2670 h^−1^ and 115 h^−1^, for passenger vehicles and city transit buses, respectively.

### Data availability

The data archive can be accessed at https://goo.gl/hyNduj.

## Electronic supplementary material

Supplementary Material
