# Supplementary material for: Disentangling vehicular emission impact on urban air pollution using ethanol as a tracer
Source: Sci Rep. 2018 Jul 16;8:10679. doi: 10.1038/s41598-018-29138-7 (PMC6048126; doi:10.1038/s41598-018-29138-7)
Supplement: Supplementary file 1 — Supplementary Material [file 41598_2018_29138_MOESM1_ESM.docx]

Disentangling vehicular emission impact on urban air pollution using ethanol as a tracer

Joel Brito, Samara Carbone, Djacinto A. Monteiro dos Santos, Pamela Dominutti, Nilmara de Oliveira Alves, Luciana V. Rizzo, Paulo Artaxo.

SUPPLEMENTARY MATERIAL

Positive Matrix Factorization

Positive Matrix Factorization (PMF) has been conducted on unit mass resolution spectra of organic species for source apportionments. Organic data matrix and error matrix are generated from ACSM analysis software. The PMF Evaluation Toolkit (PET) software is utilized to process the data ^1^. Any “weak” m/z’s (signal-to-noise ratio between 0.2 and 2) are downweighted by a factor of 2, and “bad” m/z’s (SNR smaller than 0.2) are removed^1^. The PMF solutions for the dataset has been obtained following the detailed procedure described in Zhang et al., (2011). For the dataset analyzed here, a 4-factor solution is chosen after carefully checking the quality of the fit parameter (Fig. S1), mass spectra (Fig. 5), correlation with external tracer (Fig. 4) and diurnal variability (Figures 2 and S2). Solutions with more than 4 factors depict no significant improvement resolving individual m/z’s (Fig. S2), as well as display splitting behavior of existing factors instead of providing new factors (Zhang et al., 2011). The rotational ambiguity of the 4-factor solution is examined by varying the FPEAK parameter, displaying a almost constant mass apportionment of LDV-OA and HDV-OA for a wide range of values, with FPEAK=-0.2 being chosen due to improved correlation with external tracers and mass spectra standards.

**b)**

| 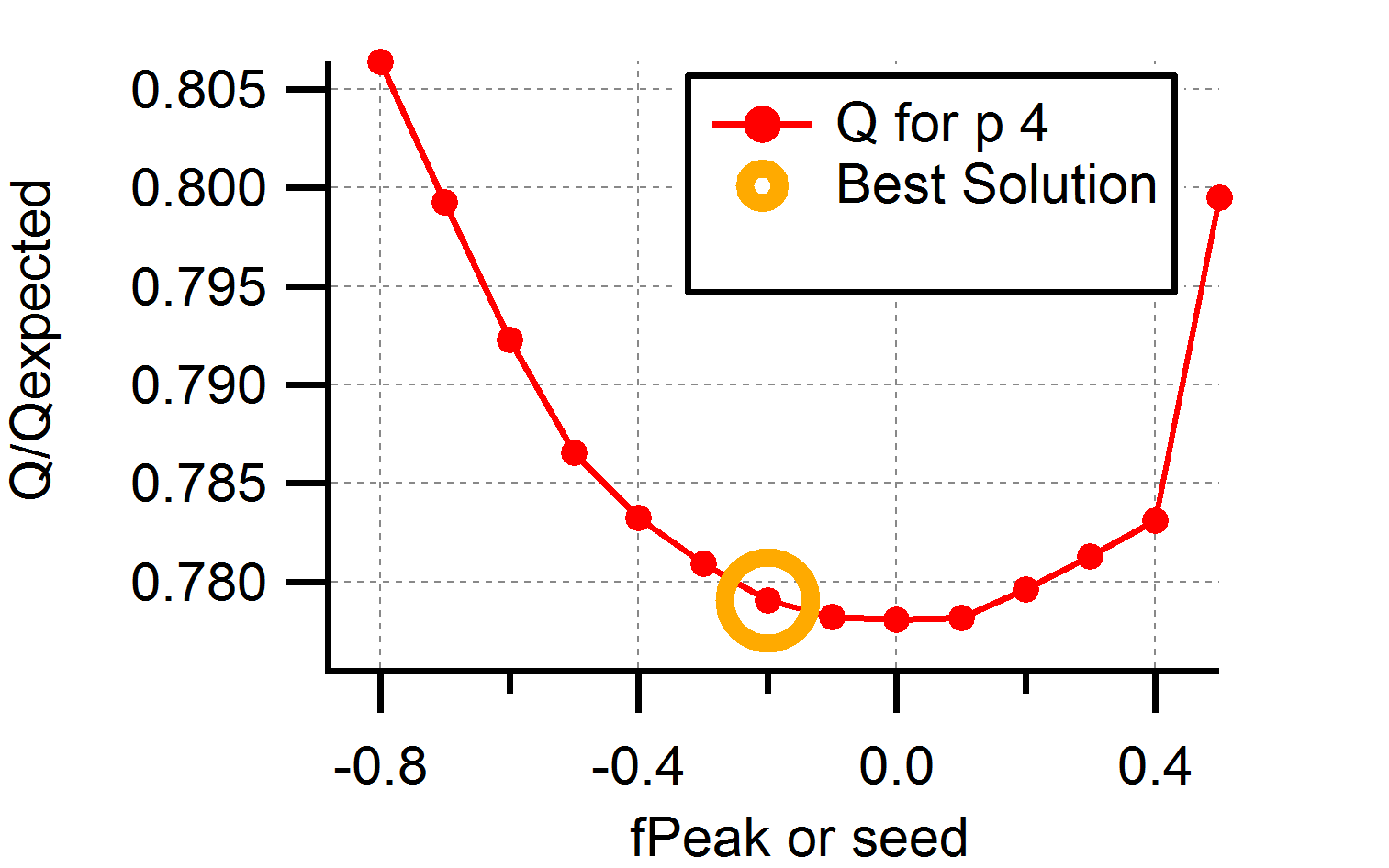  **c)**  **a)** | 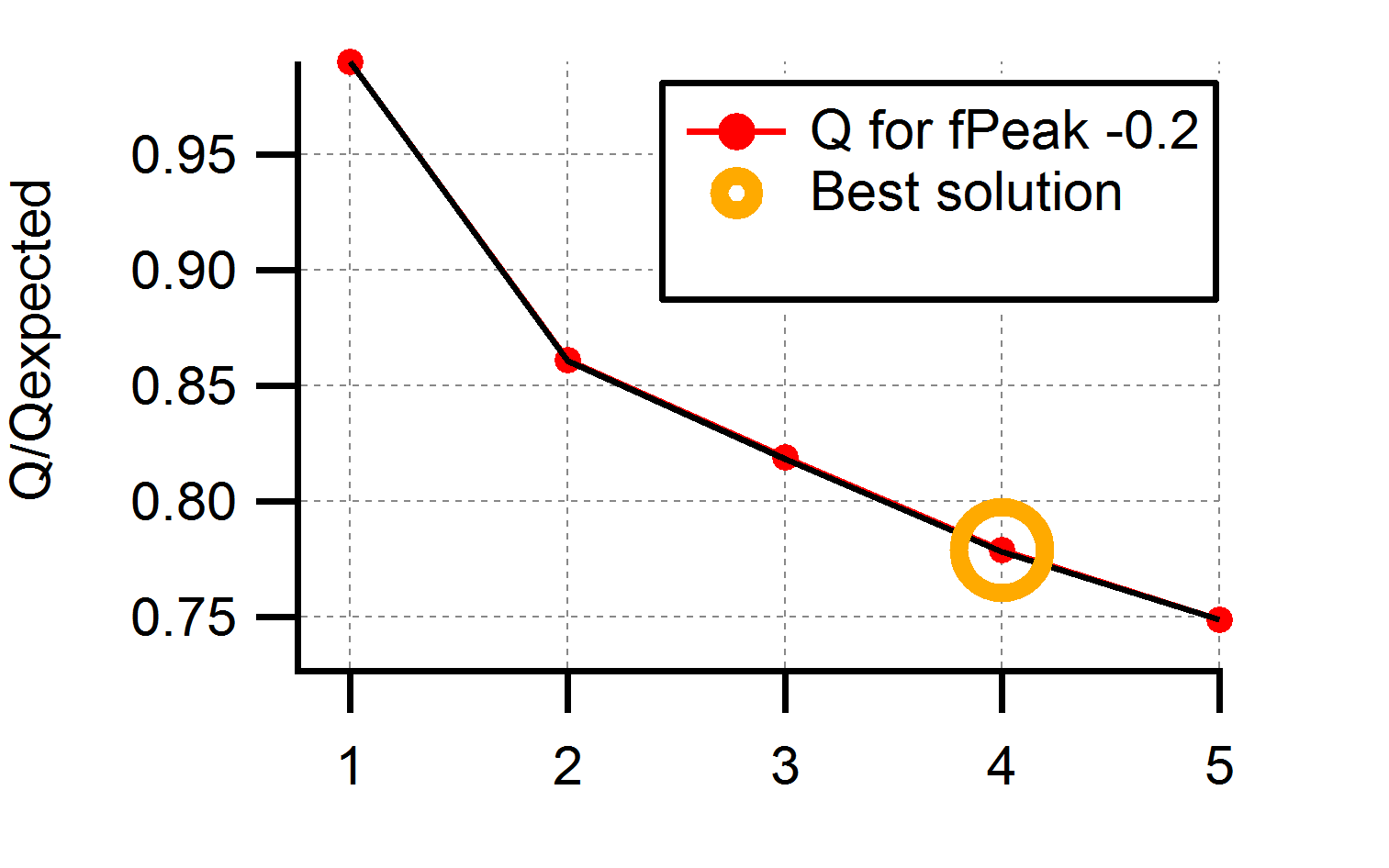 |
| --- | --- |
| **Factor #1 (HDV-OA)**  **Factor #2 (LDV-OA)**  **Factor #3 (OOA-I)**  **Factor #4 (OOA-II)** 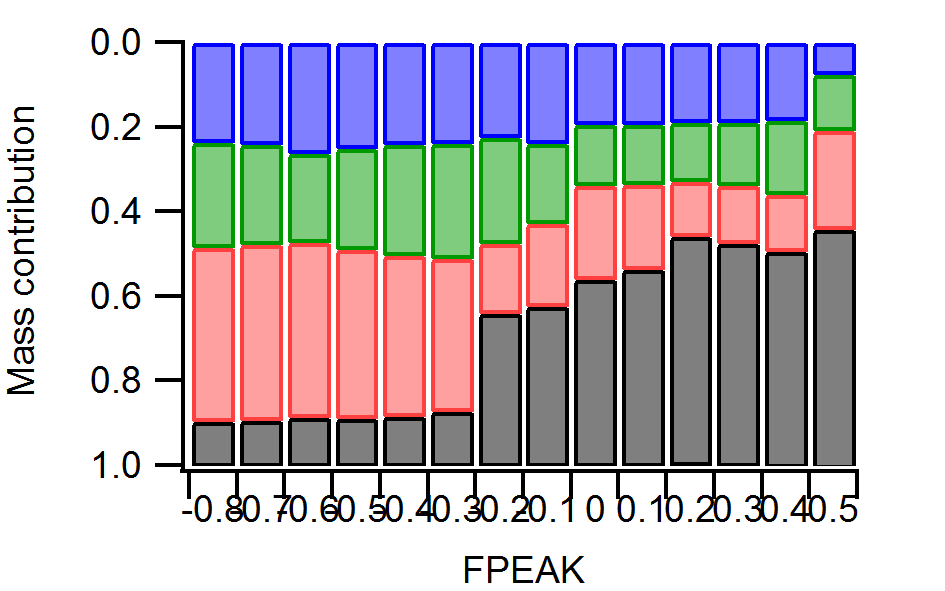 | **d)** 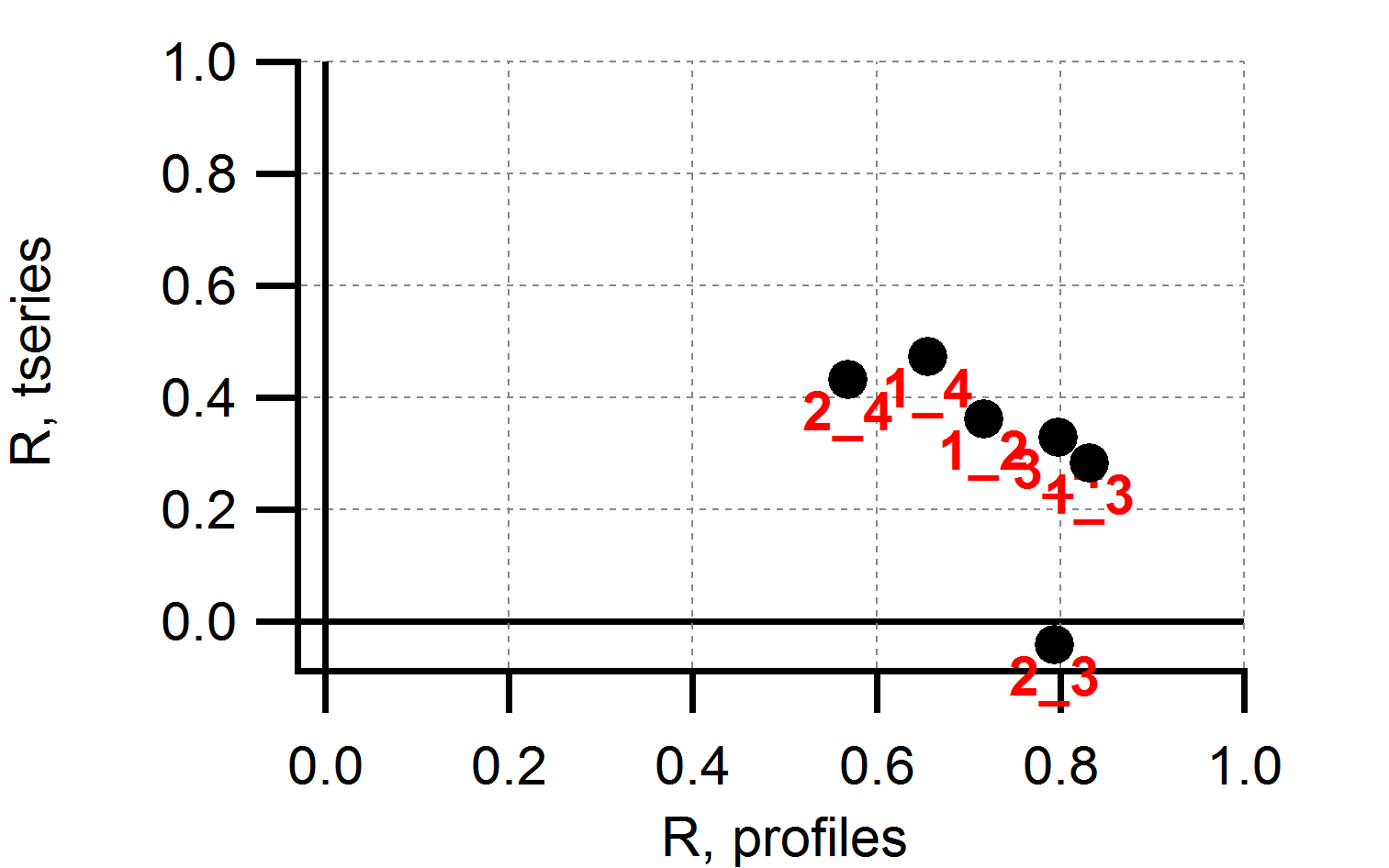 |
| **e)** 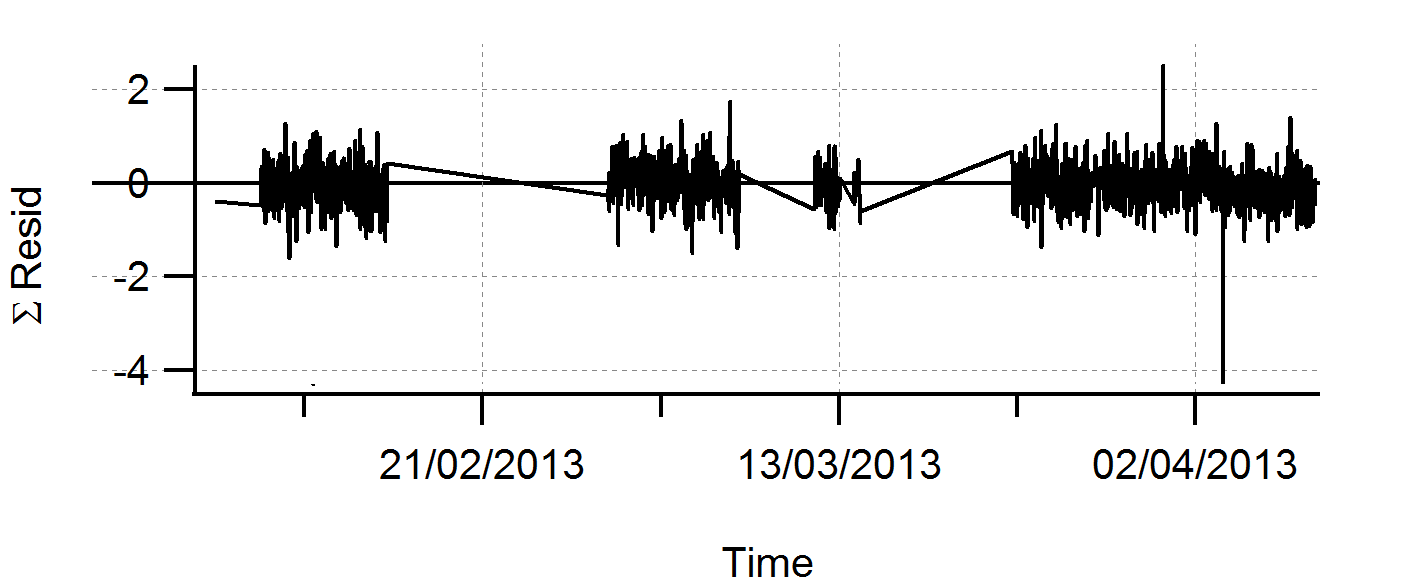 | **f)** 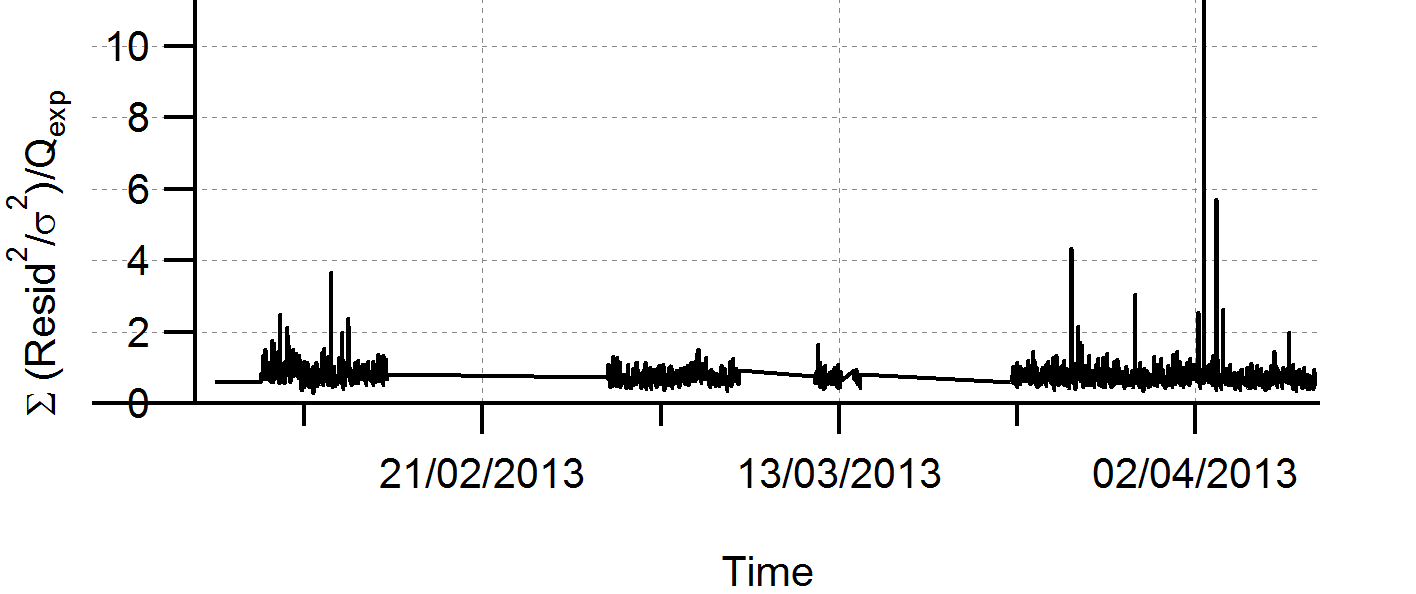 |
| **g)** 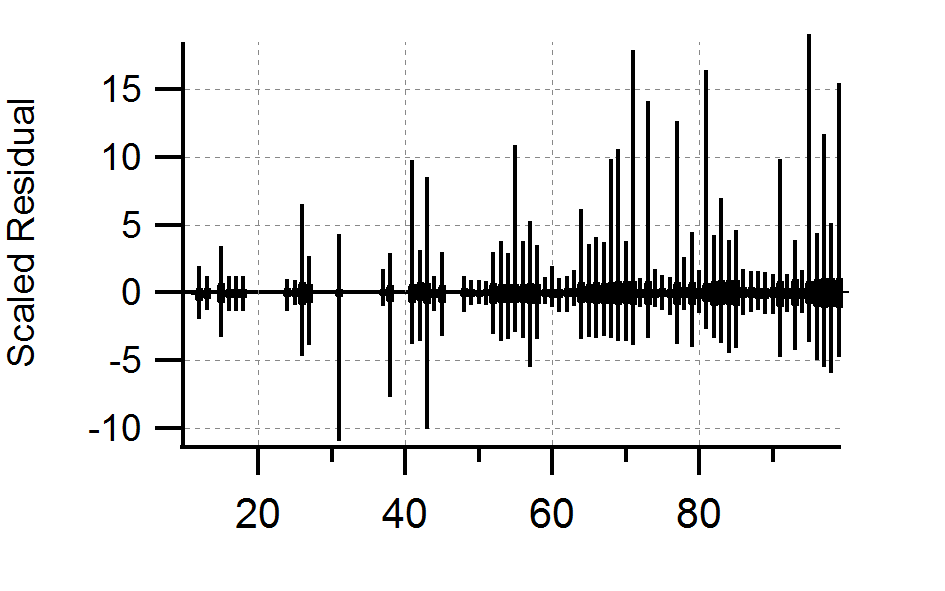 |  |

**Fig. S1.** Summary of key diagnostic plots of the PMF results. (a) Q/Qexp as a function of number of factors. (b) Q/Qexp as a function of FPEAK for the 4-factor solution. (c) Mass fraction of PMF factors as a function of FPEAK. (d) Correlations of time series and mass spectra among PMF factors. (e) Variations of the residual (= measured - reconstructed) of the least-square-fit as a function of time. (f) The Q/Qexp for each point as a function of time. (g) The Scaled Residuals for each m/z.

Weekend diurnal variability

The plot below shows same parameters as Fig 2 (main text) for weekend.


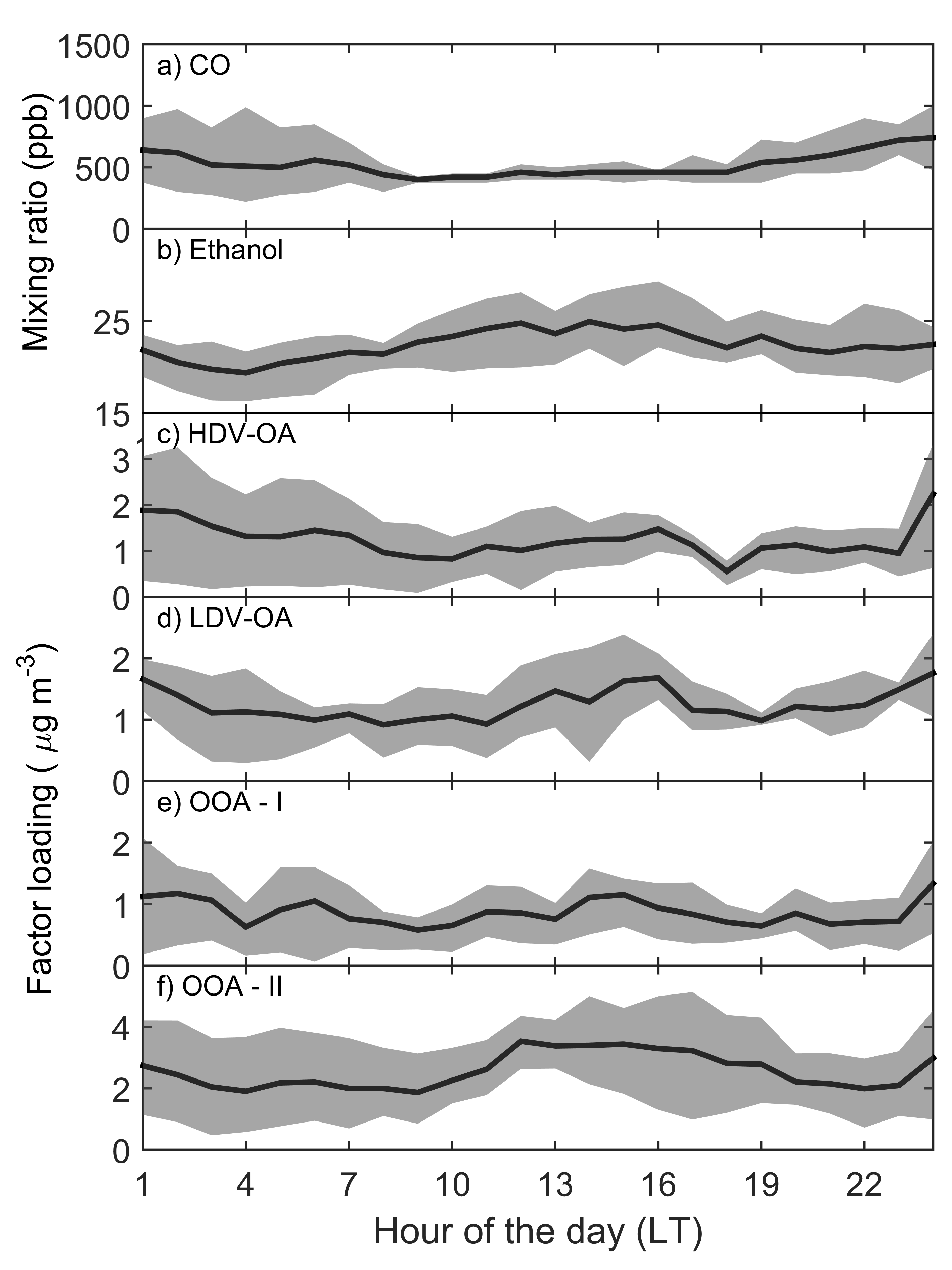


**Figure S2.** Weekend diurnal profile of CO (a), ethanol (b), HDV factor (c), LDV factor (d), OOA-I factor (e), OOA-II factor (f). Black line represents median and grey area the interquartile range.

Multiple Linear Regression results

**Table S1.** Adjusted R^2^ and fit parameters of the Multiple Linear Regression results between PMF factors and species of interest.

|  | Adj. R^2^ | Normalized Mean Bias (%) | OOA-II | OOA-I | LDV-OA | HDV-OA |
| --- | --- | --- | --- | --- | --- | --- |
| CO | 0.84 | 8.0 | 0.002 (0.010) | 0.051 (0.036) | 0.280 (0.012) | 0.145 (0.017) |
| NO | 0.62 | 9.5 | -6.271 (0.995) | -1.730 (3.434) | 17.398 (1.217) | 15.765 (1.618) |
| Ozone | 0.79 | -21.2 | 6.609 (0.479) | 5.969 (1.583) | 0.435 (0.507) | -4.473 (0.707) |
| Acetaldehyde | 0.90 | 7.1 | 0.353 (0.039) | 0.575 (0.137) | 1.119 (0.047) | 0.347 (0.064) |
| Benzene | 0.90 | 5.0 | -0.015 (0.008) | 0.106 (0.028) | 0.255 (0.010) | 0.180 (0.013) |
| Toluene | 0.83 | 4.7 | -0.089 (0.032) | 0.210 (0.111) | 0.811 (0.039) | 0.604 (0.052) |
| PM_10_ | 0.92 | 4.1 | 3.963 (0.317) | 3.480 (1.113) | 8.282 (0.387) | 4.476 (0.521) |
| PM_1_ | 0.95 | 1.3 | 2.000 (0.108) | 1.995 (0.378) | 2.207 (0.131) | 2.726 (0.177) |
| N | 0.79 | 12.3 | 219.336 (262.618) | 5132.916 (924.445) | 4496.677 (318.442) | 1002.329 (417.375) |
| Nitrate | 0.69 | -10.4 | 0.165 (0.023) | 0.152 (0.082) | -0.065 (0.028) | 0.396 (0.038) |
| Sulphate | 0.67 | 5.8 | 0.688 (0.053) | 0.447 (0.186) | 0.148 (0.065) | 0.015 (0.087) |
| BC | 0.90 | 2.6 | -0.147 (0.040) | 0.185 (0.139) | 1.219 (0.048) | 1.189 (0.065) |

**References**

1. Ulbrich, I. M., Canagaratna, M. R., Zhang, Q., Worsnop, D. R. & Jimenez, J. L. Interpretation of organic components from Positive Matrix Factorization of aerosol mass spectrometric data. *Atmos. Chem. Phys.* **9,** 2891–2918 (2009).

2. Zhang, Q. *et al.* Understanding atmospheric organic aerosols via factor analysis of aerosol mass spectrometry: A review. *Anal. Bioanal. Chem.* **401,** 3045–3067 (2011).
